# Bacteria Found in Perspiration of Man

**Published:** 1876-04

**Authors:** 


Bacteria Found in the Perspiration of Man.—Eberth, of Zurich, Switzerland, has found, says the *Medical Record*, by the aid of the microscope, in the sweat of the face some corpuscles which he considered as bacteria. This view became confirmed when he examined the axilla, breast, and inner side of the thigh of several persons in a state of perspiration. The sweat of these parts contained nearly always enormous numbers of bacteria. In most cases they originated from minute bodies found upon the hairs in the mentioned regions, forming little nodules on them, and giving them a grayish or a brick color. They were recognized by the author as accumulations of micrococci. They may rapidly increase in number, are smaller than the diphtherial micrococci, and are nearly indifferent to re-agents (concentrated acids, alkalies, alcohol, ether, chloroform). Iodine colors them yellow. The vegetation of bacteria on the hairs may be observed in cases where they are changed already, beginning in places which have clefts between their cells. The vegetation occupies large spaces, especially in the direction of the longest diameter of the hair.

Dr. Elberth observed a mycelium and micrococci, and thinks that the latter are the fruits of the former. Other investigators observed colored sweat, red and blue, which contained micrococci. It was difficult to decide in these cases if the coloring matter was adherent to the micrococci, or if it was a product of the vegetation.

